# Multi-Wavelength Selective and Broadband Near-Infrared Plasmonic Switches in Anisotropic Plasmonic Metasurfaces

**DOI:** 10.3390/nano13243141

**Published:** 2023-12-15

**Authors:** Yan Li, Yaojie Zhou, Qinke Liu, Zhendong Lu, Xiao-Qing Luo, Wu-Ming Liu, Xin-Lin Wang

**Affiliations:** 1 School of Nuclear Science and Technology, University of South China, Hengyang 421001, China; 2 School of Electrical Engineering, University of South China, Hengyang 421001, China; 3 Beĳing National Laboratory for Condensed Matter Physics, Institute of Physics, Chinese Academy of Sciences, Beĳing 100190, China; 4 Hunan Province Key Laboratory for Ultra-Fast Micro/Nano Technology and Advanced Laser Manufacture, School of Mechanical Engineering, University of South China, Hengyang 421001, China

**Keywords:** metasurface, multi-wavelength selective, Fano resonances, plasmonic switches

## Abstract

Anisotropic plasmonic metasurfaces have attracted broad research interest since they possess novel optical properties superior to natural materials and their tremendous design flexibility. However, the realization of multi-wavelength selective plasmonic metasurfaces that have emerged as promising candidates to uncover multichannel optical devices remains a challenge associated with weak modulation depths and narrow operation bandwidth. Herein, we propose and numerically demonstrate near-infrared multi-wavelength selective passive plasmonic switching (PPS) that encompasses high ON/OFF ratios and strong modulation depths via multiple Fano resonances (FRs) in anisotropic plasmonic metasurfaces. Specifically, the double FRs can be fulfilled and dedicated to establishing tailorable near-infrared dual-wavelength PPS. The multiple FRs mediated by in-plane mirror asymmetries cause the emergence of triple-wavelength PPS, whereas the multiple FRs governed by in-plane rotational asymmetries avail the implementation of the quasi-bound states in the continuum-endowed multi-wavelength PPS with the ability to unfold a tunable broad bandwidth. In addition, the strong polarization effects with in-plane anisotropic properties further validate the existence of the polarization-resolved multi-wavelength PPS. Our results provide an alternative approach to foster the achievement of multifunctional meta-devices in optical communication and information processing.

## 1. Introduction

The optical metasurfaces that are ultrathin planar nanostructures encompassing periodic arrangement of subwavelength metal or dielectric unit cells have been considered as potential candidates for the study of nanoscale light-matter interaction, enabling an alternative approach to control the amplitude, phase, and polarization of the light [1,2,3,4,5,6,7,8,9]. Polarization, one of the most important characteristics of light, offers great possibilities for the development of optical applications and techniques [10,11]. The traditional counterpart to advanced controlling polarization suffers from stringent requirements on material features and limited optical performance. The plasmonic metasurfaces with polarization-resolved characteristics can exhibit anisotropic optical responses to light waves by tailoring the in-plane asymmetric configuration (i.e., mirror or rotational asymmetries) of the unit cells [12,13,14,15]. As predicted, the anisotropic plasmonic metasurfaces, which can elicit the modulation of the polarization state of transmitted and reflected light, have sparked considerable interest in polarization conversion [16,17,18], plasmon hybridization engineering [19], bound states in the continuum (BICs) [20,21], and so on.

Recently, it is notable that plasmonic Fano resonance (FR) is a fundamentally interesting phenomenon derived from the interference between broad superradiant and narrow subradiant modes, thus leading to strong field enhancement and wavelength selectivity with sharp asymmetric spectral ling shape. This can enable the FR-based applications, such as optical sensing [22,23,24,25,26], switching [26,27,28,29], modulators [30,31,32], filter [33], and BIC-supporting metadevices [20,34,35]. The multiple FRs, which can incur multi-wavelength selectivity at several spectral positions, are capable of outperforming the single plasmonic FR and can offer a variety of new attractive features in plasmonic metasurfaces due to being more sensitive to the structural parameters and the electromagnetic parameters of surrounding media [36]. Following this, the multiple plasmonic FRs have been theoretically proposed and experimentally observed in various systems, including split nanorings [37,38], symmetry breaking plasmonic system [39,40,41], and plasmonic clusters [42,43,44,45], etc. Therein, the plasmonic multiple FRs generally appear in plasmonic multiparticle systems, complex and multilayer nanostructures. Only a few studies reveal characteristic distinctive amplitude-modulation flexibility and multi-wavelength selectivity for information transmitting and processing. Furthermore, the practical applications of multiple FR-based devices are restricted by the challenges associated with the fabrication of multiparticle and complex systems. In addition, the plasmonic metasurfaces with split ring unit cells can disclose anisotropic resonant response to light waves by adjusting their asymmetric geometrical configurations. It is also worth emphasizing that the linearly polarized light can render the artificial optical anisotropy to be strongest. Therefore, with the view of elucidating multi-wavelength selective optical switching with strong modulation depths and broad operation bandwidth, the multiple FRs-empowered anisotropic plasmonic metasurfaces still need to be further explored.

In this work, we propose and numerically demonstrate near-infrared multi-wavelength selective passive plasmonic switching (PPS), possessing high ON/OFF ratios and strong modulation depths, via multiple Fano resonances (FRs) in anisotropic plasmonic metasurfaces. It is shown that the double FRs, boosting the presence of double bonding mode and anti-bonding mode, as well as the degenerate resonance mode, can be fulfilled and dedicated to establishing tailorable near-infrared dual-wavelength PPS by properly adjusting the orientation of the incident light and the related structural parameters of the unit cells. Leveraging on the in-plane mirror asymmetries, the multiple FRs-based triple-wavelength PPS can be accomplished. Empowered by the in-plane rotational asymmetries, the quasi-bound states in the continuum-embedded multiple FRs can be harnessed to achieve multi-wavelength PPS associated with a tunable broad bandwidth. In addition, the strong polarization effects with in-plane anisotropic properties provide further evidence for unveiling the polarization-resolved multi-wavelength PPS. Our results can be utilized to facilitate multi-wavelength selective and anisotropic meta-devices for optical communication and information processing.

## 2. The Model of the Hybrid Metasurface

**Figure 1:**
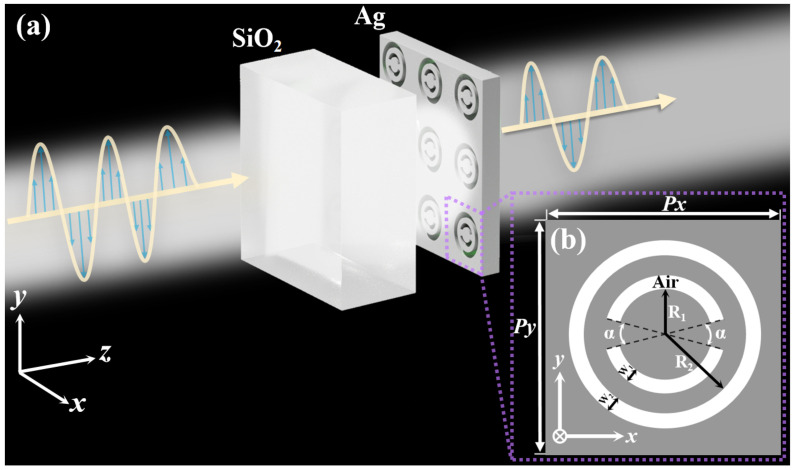
(**a**) Schematic diagram of the hybrid plasmonic metasurface consisting of periodic silver (Ag) thin-film arrays perforated with concentric double-split ring aperture (DSRA) and circular-ring aperture (CRA) unit cells. The propagation of the incident plane wave is along the positive Z-direction from the side of the quartz substrate. The Ag film is stuck to the quartz substrate without any air gap. (**b**) The zoomed top-view of the hybrid metasurface in the X-Y plane. $P_{x}$ and $P_{y}$ are the periodic lengths of the unit cell in X- and Y-directions, respectively. The gray and white parts respectively represent the Ag film and the air. $R_{1}$ ($R_{2}$) and $W_{1}$ ($W_{2}$) depict the inner radius and the width of the DSRA (CRA) unit cell, respectively. α delineates the opening angle of DSRA, where the in-plane mirror symmetries are preserved along the X- and Y-axis, respectively.

Herein, we propose a hybrid metasurface that encompasses periodic silver (Ag) thin-film arrays, as shown in Figure 1a, with concentric double-split ring aperture (DSRA) and circular-ring aperture (CRA) unit cells. The thickness and dielectric constant of the substrate (quartz) are set as 225 nm and 2.25 [46], respectively. The thickness of the Ag film that is adopted to construct nanoscale-aperture arrays on the quartz substrate is set as 50 nm [47]. The dielectric constant of Ag film can be given by the improved Drude model [$\epsilon \left(\omega \right)=\epsilon_{\infty }-\omega_{p}^{2}/\left(\omega^{2}+i\gamma \omega \right)$] [48], where the bulk plasmon frequency $\omega_{p}=1.3388\times 10^{16}\;\mathrm{rad}/s$, the damping rate $\gamma =7.07592\times 10^{13}\;\mathrm{rad}/s$, and the dielectric constant of infinite frequency $\epsilon_{\infty }=3.36174$, respectively. The incident plane wave, under normal incidence and from the side of the quartz substrate, propagates along the positive Z-direction. The surrounding medium encircling the hybrid metasurface is air, where the refractive index equals 1.0. Figure 1b exhibits the zoomed top-view of the unit cell of the hybrid metasurface in the X-Y plane. $P_{x}$ and $P_{y}$ are the periodic lengths of the periodic nanoscale aperture arrays in the X- and Y-direction, respectively. The gray area depicts the Ag film, while the white area denotes the air. $R_{1}$ ($R_{2}$) and $W_{1}$ ($W_{2}$) are the inner radius and the width of the DSRA (CRA) unit cell, respectively. α represents the opening angle of DSRA, thus leading to the in-plane mirror symmetries being preserved along the X- and Y-direction. Additionally, the optical transmittance properties of the hybrid metasurface are conducted using the three-dimensional finite-difference time-domain method. A cubic Yee cell with a uniform cubic grid (Δx=Δy=Δz=2nm) and a time step (Δt=1as) are adopted to sustain the numerical stability condition. The X- and Y-directions of the hybrid metasurface are chosen as the periodic boundary conditions, while the perfectly matched layers are considered as the boundary conditions on the front and rear sides of the Z-direction. After setting up the initial value and boundary conditions, the electric and magnetic fields are sampled alternately in each half-time step, and the computation is conducted iteratively. Moreover, a monitoring surface is positioned 200 nm above the Ag film in order to enable real-time monitoring of the dispersion of electromagnetic fields.

## 3. Plasmonic Double Fano Resonances in a Hybrid Metasurface

**Figure 2:**
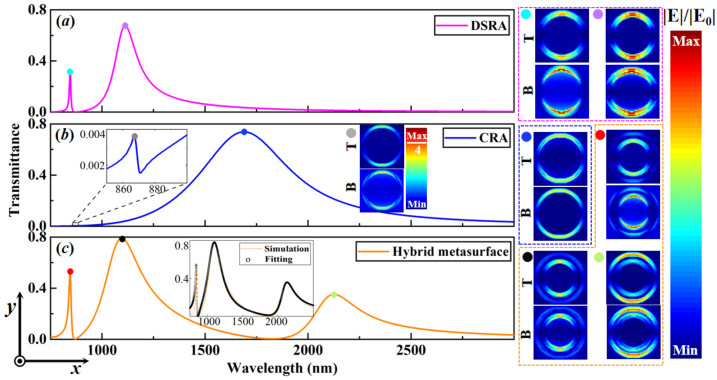
The transmittance spectra of the periodic Ag thin-film arrays with (**a**) individual DSRA unit cells, (**b**) individual CRA unit cells, and (**c**) the hybrid metasurface. The related electric field distributions of the transmittance spectra at the labeled wavelength are shown on the right side, which contains both the top (T) panel (at the Ag/air interface) and the bottom (B) panel (at the Ag/quartz interface). The right inset of (**b**) shows the electric field distribution of the transmittance spectrum at the grey dot. While the inset of (**c**) shows the corresponding simulation (orange curve) and fitting (black circle) of the related transmittance spectrum, respectively. The geometrical parameters of the unit cell of the hybrid metasurface are given as follows: $P_{x}=P_{y}=600\;\mathrm{nm}$, $R_{1}=100\;\mathrm{nm}$, $W_{1}=30\;\mathrm{nm}$, $R_{2}=150\;\mathrm{nm}$, $W_{2}=25\;\mathrm{nm}$, α=40°.

Next, we study the plasmonic double FRs, as illustrated in Figure 2c, in a hybrid metasurface that is composed of concentric DSRA and CRA unit cells, which is different from two metasurfaces only including DSRA unit cells in Figure 2a and CRA unit cells in Figure 2b, respectively. The incident polarization direction is along the Y-direction. The periods ($P_{x}$ and $P_{y}$) of the unit cells of the three types of metasurfaces all are set to 600 nm. The inner radius $R_{1}$ ($R_{2}$) and the width $W_{1}$ ($W_{2}$) of the DSRA (CRA) unit cell are set as 100 nm (150 nm) and 30 nm (25 nm), respectively. The opening angle α of the individual DSRA and hybrid metasurfaces are set as 40°, where both the in-plane (X-Y plane) mirror symmetries are preserved. The transmittance spectrum of the metasurface with individual DSRA unit cells, as displayed in Figure 2a, exhibits an asymmetric lineshape as a result of the interaction between the quadrupole ($Q_{\mathrm{DSRA}}$) and electric dipole ($D_{\mathrm{DSRA}}$) modes [49,50], thus leading to the appearance of bonding ($D_{\mathrm{DSRA}}+Q_{\mathrm{DSRA}}$), labeled as a purple dot, and anti-bonding modes ($D_{\mathrm{DSRA}}-Q_{\mathrm{DSRA}}$), marked as a light-blue dot. Despite plasmonic hybridization of wave functions between $D_{\mathrm{DSRA}}$ and $Q_{\mathrm{DSRA}}$ modes [49], the former is more efficiently excitated owing to the specific polarization state of the incident light. This process gives rise to enhancing the electric field distribution along the incident polarization direction. Therein, the top (T) and bottom (B) panels show the electric field distributions of the *Ag/air* and *Ag/quartz* interfaces, respectively. Likewise, for the case of the metasurface with individual CRA unit cells, as shown in Figure 2b, the transmittance spectrum shows the bonding ($D_{\mathrm{CRA}}+Q_{\mathrm{CRA}}$) and anti-bonding modes ($D_{\mathrm{CRA}}-Q_{\mathrm{CRA}}$). The bonding mode, labeled as a blue dot, reveals more dipolar-like resonance, while the anti-bonding mode, marked as a gray dot, unfolds dipolar and quadrupolar resonances due to the dielectric properties of the local environment, which can also be verified from the bottom panel of the enhanced electric field distribution in the inset of Figure 2b. As shown in Figure 2c, once there exists spectral overlap of the transmittance spectra between the two pairs of bonding and anti-bonding modes, it is of great interest to notice that only three new hybridized resonance modes are obtained. This process results in the realization of plasmonic double FRs induced by the double bonding and anti-bonding modes, as well as the degenerate resonance mode. The *double bonding* mode, marked as a green dot, arises from the interaction between the bonding state ($D_{\mathrm{DSRA}}+Q_{\mathrm{DSRA}}$) and the bonding ($D_{\mathrm{CRA}}+Q_{\mathrm{CRA}}$) modes, which showcases more dipolar-like resonance. Whereas the *double anti-bonding* mode, which is labeled as a red dot, originates from the interaction between the anti-bonding state ($D_{\mathrm{DSRA}}-Q_{\mathrm{DSRA}}$) and the anti-bonding ($D_{\mathrm{CRA}}-Q_{\mathrm{CRA}}$) modes, uncovering dipolar and quadrupolar resonances. The degenerate resonance mode, marked as a black dot, stems from the *double bonding* mode [($D_{\mathrm{DSRA}}-Q_{\mathrm{DSRA}}$) + ($D_{\mathrm{CRA}}-Q_{\mathrm{CRA}}$)] or *double anti-bonding* mode [($D_{\mathrm{DSRA}}+Q_{\mathrm{DSRA}}$) − ($D_{\mathrm{CRA}}+Q_{\mathrm{CRA}}$)], which unravels the dipolar and quadrupolar resonances that can be approximately treated as ($D_{\mathrm{DSRA}}-Q_{\mathrm{CRA}}$) [51,52]. These behaviors can also be more effectively validated by the bottom panels of the electric field distribution in the inset of Figure 2.

To further understand the underlying physical mechanism of the plasmonic double FRs, the multimode interference coupled mode theory (MICMT), a simple and effective tool, can be used to unveil them in the hybrid metasurface [53]. It has been proven that the transmittance spectra of the multiple resonance modes are strongly influenced by the coupling phases among them. In doing so, the MICMT fundamental equations can be given as,

(1) $$\begin{array}{cc} S_{n,a}^{I} & =\sum_{n=0}^{N}\gamma_{n,a}e^{i\varphi_{n,a}}S_{a}^{I},\;\;S_{n,b}^{I}=\sum_{n=0}^{N}\gamma_{n,b}e^{i\varphi_{n,b}}S_{b}^{I}, \end{array}$$

(2) $$\begin{array}{cc} S_{a}^{O} & =-S_{a}^{I}+\sum_{n}\kappa_{n,a}^{*}c_{n},\;\;S_{b}^{O}=-S_{b}^{I}+\sum_{n}\kappa_{n,b}^{*}c_{n}, \end{array}$$

(3) $$\begin{array}{cc} \kappa_{n,a} & =\sqrt{2/\tau_{n,a}}e^{i\theta_{n,a}},\;\;\kappa_{n,b}=\sqrt{2/\tau_{n,b}}e^{i\left(\theta_{n,a}-\phi_{n}\right)}, \end{array}$$

(4) $$\begin{array}{cc} \sum_{n=0}^{N}\frac{dc_{n}}{dt} & =\left(-i\omega_{n}-\sum_{j=0}^{2}\frac{1}{\tau_{n,j}}\right)c_{n}+\kappa_{n,a}S_{a}^{I}+\kappa_{n,b}S_{b}^{I}, \end{array}$$

where $S_{a(b)}^{I(O)}$ denote the field amplitudes for the input (I) and output (O) ports of a and b, respectively. $\gamma_{n,a}e^{i\varphi_{n,a}}$ and $\gamma_{n,b}e^{i\varphi_{n,b}}$ represent the normalized coefficients with $\gamma_{n,a}=\gamma_{n,b}\approx 1$. $\varphi_{n,k}$, $\theta_{n,k}$, and $\kappa_{n,k}\;\left(k=a,b\right)$ ($\kappa_{n,k}^{*}$ are the related complex conjugate terms) are the complex amplitude phase, coupling phases, and coupling coefficients of the $n^{th}$ resonance mode, respectively. $\phi_{n}$ is the phase difference between the output and input ports of the $n^{th}$ resonance mode. $\tau_{n,j}$ depict the decay time of the coupling between the resonance mode and environment, respectively. $c_{n}$ and $\omega_{n}$ are the field amplitude and resonant frequency of the resonance mode, respectively. $\tau_{n,0}$ is the decay time of internal loss of the $n^{th}$ resonance mode. Based on the single-input case, the $S_{b}^{I}$ can be set to 0. Thus, the transmittance of the hybrid metasurface can be written as,

(5) $$T={\left|\frac{S_{b}^{O}}{S_{a}^{I}}\right|}^{2}={\left|\sum_{n=1}^{N}\frac{2e^{i\varphi_{n}}}{-i\left(\omega -\omega_{n}\right)\tau_{n}+2+\tau_{n0}}\right|}^{2},$$

where $\tau_{n0}=\tau_{n}/\tau_{n,0}$ and $\varphi_{n}=\varphi_{n,a}+\theta_{n,a}-\theta_{n,b}+\phi_{n}$ is the total coupling phase difference for resonance modes of the plasmonic double FRs, i.e., the double bonding and anti-bonding modes, as well as the degenerate resonance mode. By using the MICMT, the fitting (black circle) agrees well with the numerical simulation (orange curve), as shown in the inset of Figure 2c. The fitting parameters of the plasmonic double FRs were achieved as follows: N=3, $\omega_{1}=3.5384\times 10^{14}\;\mathrm{rad}/s$, $\omega_{2}=2.7305\times 10^{14}\;\mathrm{rad}/s$, $\omega_{3}=1.4306\times 10^{14}\;\mathrm{rad}/s$, $\tau_{1}=566\;\mathrm{fs}$, $\tau_{2}=68\;\mathrm{fs}$, $\tau_{3}=383.6\;\mathrm{fs}$, $\tau_{1,0}=1083\;\mathrm{fs}$, $\tau_{2,0}=635\;\mathrm{fs}$, $\tau_{3,0}=478\;\mathrm{fs}$, $\varphi_{1}=0.2$, $\varphi_{2}=1.1$, and $\varphi_{3}=2.1$.

## 4. Dual-Wavelength Plasmonic Switches via Double Fano Resonances

**Figure 3:**
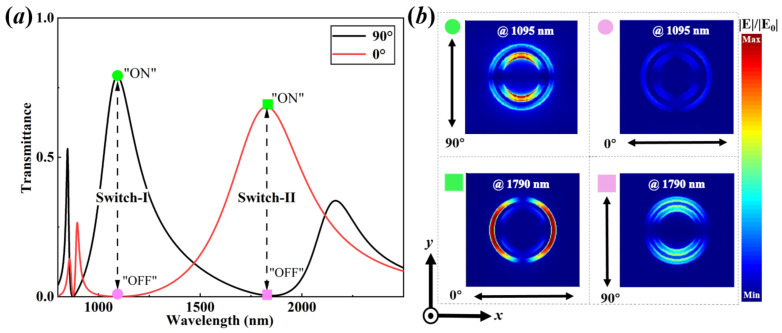
The plasmonic double FRs-induced near-infrared dual-wavelength switches in the hybrid plasmonic metasurface. (**a**) The polarization-sensitive dual-wavelength passive plasmonic switches (Switch-I and Switch-II) with ON/OFF ratio being 28.81 dB and 20.76 dB at the wavelength of 1095 nm and 1790 nm, respectively. (**b**) The electric field distributions with related polarized angles of the incident light at the switching points of (**a**). The parameters are the same as Figure 2.

It has been proved that the peak and position of the plasmonic FRs can be tailored by varying the related geometric parameters of the metasurfaces, which can also trigger the presence of polarization-dependent transmittance spectra in the near-infrared regions. Thereafter, the maximal and minimal transmittance spectra governed by the plasmonic FRs can be designed as the “ON” and “OFF” states of the plasmonic switches at the relative wavelengths, respectively. In this context, for the central wavelengths of 1095 nm and 1790 nm, the polarization-sensitive transmittance spectra can be achieved by modulating the polarized angle of the incident light, as shown in Figure 3a. Thus, the ON/OFF ratio (η) can be defined as [54,55,56,57]:

(6) $$\eta =10\log_{10}\left(\frac{T_{\mathrm{ON}}}{T_{\mathrm{OFF}}}\right),$$

with $T_{\mathrm{ON}}$ and $T_{\mathrm{OFF}}$ being the “ON” and “OFF” states of the switching points, respectively. As shown in Figure 3a, the “ON” and “OFF” states of the Switch-I and Switch-II are presented at the central wavelengths of 1095 nm and 1790 nm, respectively. For the case of the Switch-I, the “ON” state that originates from the degenerate resonance mode can be obtained at the second peak of the longitudinally polarized double FRs (see the green dot in Figure 3a), where $T_{\mathrm{ON}}=0.79046$ and the incident light is vertically polarized to the DSRA unit cells (along the Y-direction). While the “OFF” state can be achieved at the second dip of the transversely polarized double FRs (see the pink dot in Figure 3a). Therein, $T_{\mathrm{OFF}}=0.00104$ and the incident light is horizontally polarized to DSRA unit cells (along the X-direction). Hence, the ON/OFF ratio (η) govern by Equation (6) can be acquired as 28.81 dB with the corresponding modulation depth [($1-T_{\mathrm{OFF}}/T_{\mathrm{ON}})\times 100\%$] being 99.87%. Simultaneously, the electric field distribution, as shown in Figure 3b, indicates that the degenerate resonance mode ($D_{\mathrm{DSRA}}-Q_{\mathrm{CRA}}$) is mostly distributed in the DSRA unit cells for the “ON” state, but there is no apparent electric field distribution for the “OFF” state. For the case of the Switch-II, the “ON” state can be attained at the third peak of the transversely polarized double FRs (see the green square in Figure 3a) with $T_{\mathrm{ON}}=0.68794$. Whereas the “OFF” state can be accomplished at the second dip of the longitudinally polarized double FRs (see the pink square in Figure 3a) with $T_{\mathrm{OFF}}=0.00577$. As predicted, the ON/OFF ratio (η) and the modulation depth can be achieved as 20.76 dB and 99.16%, respectively. Likewise, with respect to the case of the electric field distributions of the “ON” and “OFF” states of the Switch-II, the CRA unit cells are obviously excited for both the former and the latter cases, while the DSRA unit cells are only excited for the latter case. These mean that novel dual-wavelength passive plasmonic switching via double FRs can be implemented in the near-infrared region, which is a compact and passive device with high ON/OFF ratios.

**Figure 4:**
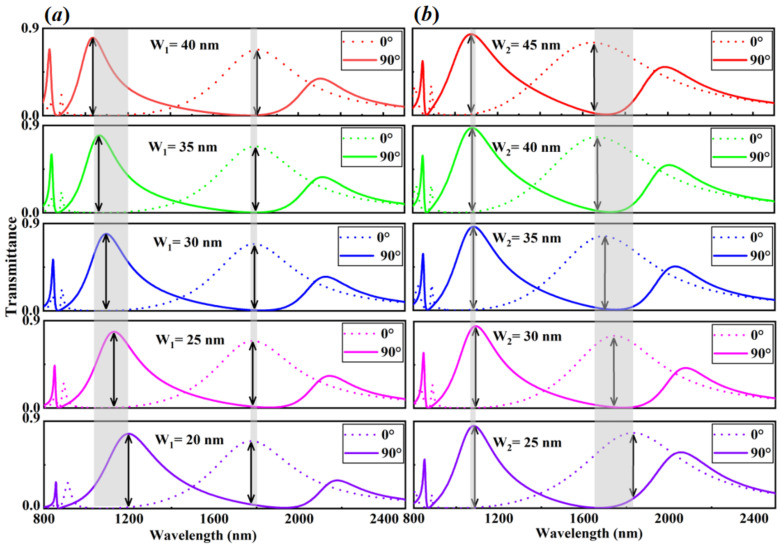
The customized dual-wavelength passive plasmonic switches induced by varying the related structural parameters. (**a**) The transmittance spectra of the hybrid metasurface with different widths of the DSRA unit cells. (**b**) The transmittance spectra of the hybrid metasurface with different widths of the CRA unit cells. The incident light is longitudinally and transversely polarized, respectively. The other parameters are the same as Figure 2.

In the following, by respectively varying the width of DSRA ($W_{1}$) and CRA ($W_{2}$) unit cells, the customized dual-wavelength passive plasmonic switches can be realized in the hybrid metasurfaces. As shown in Figure 4a, by varying the width of the DSRA unit cells ($W_{1}$) from 20 nm to 40 nm, the resonance center wavelengths of Switch-I (near 1200 nm) exhibit obvious blueshift dependence, but those of Switch-II (approximate 1800 nm) reveal weak redshift. The ON/OFF ratios of Switch-I (Switch-II) decrease from (increase to) 30.05 dB (21.59 dB) as the increase of the width of the DSRA unit cells ($W_{1}$). In the meantime, by changing the width of the CRA unit cells ($W_{2}$) from 25 nm to 45 nm, as shown in Figure 4b, the resonance center wavelengths of both Switch-I and Switch-II unfold blueshift dependence, where the latter are more prominent than the former. The ON/OFF ratios of Switch-I increase from 19.59 dB to 24.63 dB, while those of Switch-II first increase and then decrease with a maximal ratio being near 20 dB. As a consequence, these results elucidate that the Switch-I is more sensitive to the variation of the width of the DSRA unit cells ($W_{1}$) due to the change of the degenerate resonance mode that is from the longitudinally polarized double FRs, whereas the switch-II are more subject to the varied width of the CRA unit cells ($W_{2}$) owing to the alteration of the related resonance mode that is from the transversely polarized double FRs.

## 5. Triple-Wavelength Plasmonic Switches Empowered by Mirror Asymmetries

**Figure 5:**
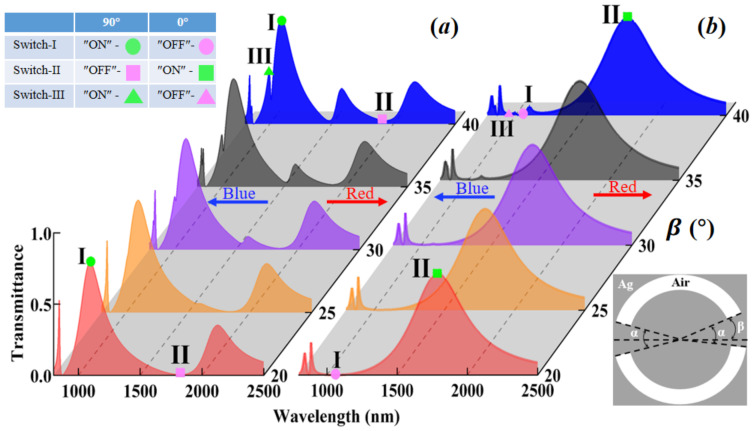
Multiple FRs-based triple-wavelength passive plasmonic switches empowered by in-plane mirror asymmetries. The color-filled transmittance spectra of the hybrid metasurfaces versus different wavelength and asymmetric angle β (which is defined as between the right side of the upper-branch of the DSRA unit cells and the X-direction, as shown in the bottom right inset), with the incident light being longitudinally polarized (90°) in (**a**) and transversely polarized (0°) in (**b**), respectively. The other parameters are the same as Figure 2.

In light of the variation of the asymmetric angle β, which is defined between the right side of the upper-branch of the DSRA unit cells and the X-direction (see the bottom right inset of Figure 5), the in-plane mirror symmetries along the X- and Y-direction of the hybrid metasurfaces are broken, thus leading to the case of mirror asymmetries. In Figure 5a, with the incident light being longitudinally polarized (90°), by altering the asymmetric angle β from 20° to 25°, there exists a new peak appearing near the redshift side of 1500 nm (see the orange-filled curve), inducing the presence of *quadruple FRs*. By increasing the asymmetric angle β to 30°, another new peak occurs near the blueshift side of 1000 nm (see the purple-filled curve), giving rise to the appearance of *quintuple FRs*. With further increasing the asymmetric angle β to 35° and 40°, the width and depth of the first and second Fano dips (near 1000 to 1500 nm) can be evidently enhanced, respectively. With regard to the incident light being transversely polarized (0°), as depicted in Figure 5b, a new peak appears near the blueshift side of 1500 nm (see the grey- and blue-filled curves), resulting in the occurrence of *triple FRs*. In this context, concerning such mirror asymmetries in the hybrid metasurfaces, the multiple FRs-based triple-wavelength passive plasmonic switches can be accomplished, where the ON/OFF ratios of Switch-I, Switch-II, and Switch-III decrease from 28.72 dB, 20.78 dB, and 16.52 dB to 10.59 dB, 11.51 dB, and 12.94 dB, with the minimal and maximal modulation depths being 91.06% and 99.87%, respectively.

## 6. Multi-Wavelength Plasmonic Switches Endowed by Rotational Asymmetries

**Figure 6:**
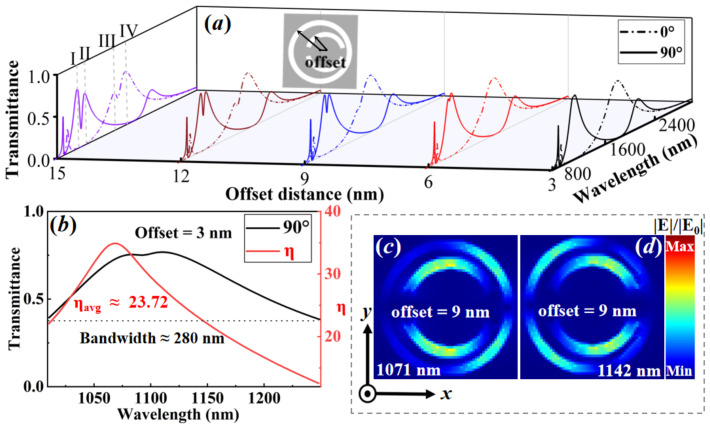
The multi-wavelength plasmonic switches enabled by breaking the C2 in-plane rotational symmetries are resorting to the variation of the transverse center offset distance between DSRA and CRA unit cells. (**a**) The transmittance spectra versus different wavelengths and offset distances, with the center of CRA unit cells being fixed. The incident light is longitudinally (90°) or transversely polarized (0°), respectively. (**b**) The tunable multi-wavelength plasmonic switches and their ON/OFF ratios η as a function of the wavelength, with the center offset distance being 3 nm and the incident light being longitudinally polarized. (**c**,**d**) The relative electric field distributions for the center offset 9 nm with the incident light being longitudinally polarized. The other parameters are the same as Figure 2.

Next, we explore the impact of breaking the in-plane rotational symmetries of C2, namely by varying the transverse center offset distance (along the positive X-direction) between DSRA and CRA unit cells, on the transmittance spectra of the hybrid metasurfaces, with the centers of CRA unit cells being fixed. The incident light is longitudinally (90°, color-solid curves) and transversely polarized (0°, color-dashed curves), respectively. As shown in Figure 6a, the transmittance spectra are strongly affected by the center offset distance, showing that the double FRs evolve to triple FRs, despite the incident light being longitudinally (90°) or transversely (0°) polarized. These behaviors elicit the appearance of *double dual-wavelength plasmonic switches* (i.e., Switch-I and Switch-II, Switch-III and Switch-IV), rendering the minimal ON/OFF ratios of these plasmonic switches as 23.72 dB, 21.21 dB, 7.34 dB, and 14.84 dB, respectively. It is interesting to notice that, for the center offset distance being 3 nm, as shown in Figure 6b, the tunable multi-wavelength plasmonic switches can be fulfilled with the bandwidth being nearly 280 nm (69 THz), when the incident light is longitudinally polarized. Therein, the corresponding ON/OFF ratios are all more than 13 dB, with almost eighty percent (54 THz bandwidth) of their ON/OFF ratios being more than 20 dB. For the relatively large value of the offset distance (e.g., 9 nm), the degenerate resonance mode that is shown in Figure 2c can be split into the *double bonding* mode [($D_{\mathrm{DSRA}}-Q_{\mathrm{DSRA}}$) + ($D_{\mathrm{CRA}}-Q_{\mathrm{CRA}}$)] and *double anti-bonding* mode [($D_{\mathrm{DSRA}}+Q_{\mathrm{DSRA}}$) − ($D_{\mathrm{CRA}}+Q_{\mathrm{CRA}}$)], which can also be evidenced by the electric field distribution in Figure 6c,d. It has been demonstrated that the small structural perturbation from breaking in the mirror or rotational symmetry of metasurfaces enables the symmetry-protected BIC, which is correlated with a vanished coupling between the resonance mode and all radiation channels of the local environment, to evolve into externally accessible quasi-BIC [20,58,59]. As expected, the results of offset distance along the positive X-direction, which are mentioned above, are identical to those along the negative X-direction only if the centers of the CRA unit cells are fixed. Thus, in contrast to the case of rotational asymmetries with center offset, the degenerate resonance mode that is from the case without center offset is in fact equivalent to a symmetry-protected plasmonic BIC [35]. This process, in turn, allows us to verify the realization of the BIC-induced plasmonic switching in Figure 3a. In addition, compared to the peak positions of the FR-based transmittance spectra in Figure 6a, it is likely to empower the generation and interplay of the plasmonic quasi-BICs for plasmonic switching, producing quasi-BICs-embedded Switch-I and Switch-II (see the color-solid curves), as well as conventional dipolar resonance-based Switch-III and Switch-IV (see the color-dashed curves).

**Figure 7:**
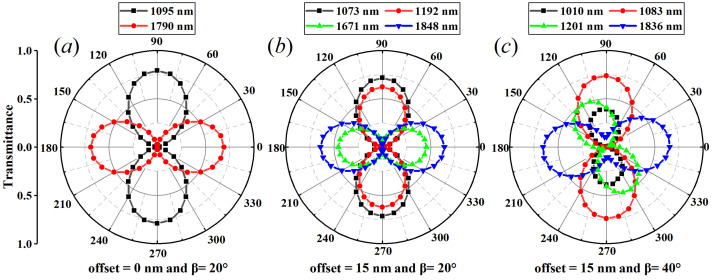
The polarization-dependent near-infrared plasmonic switching in the hybrid asymmetric metasurfaces. The polar plot of the transmittance of the related switching points (**a**) without mirror and rotational asymmetries, (**b**) without mirror asymmetries but with rotational asymmetries, and (**c**) with mirror and rotational asymmetries, respectively. The other parameters are the same as Figure 2.

To show the polarization dependence of the near-infrared plasmonic switching in the hybrid asymmetric metasurfaces, the transmittance of the related switching points can be portrayed in polar plots. The polarization angle ϑ that is defined as the angle between the polarization orientation and the positive Y-direction can be altered from 0° to 360° in a 10° step. As shown in Figure 7a, for the case of the in-plane mirror and rotational symmetries (i.e., the center offset distance equals zero and α=2β=40°), the transmittance at 1095 nm (marked with black squares) reaches a maximum value for ϑ=90° (ϑ=270°) and a minimum value for ϑ=0° (ϑ=180°). Whilst the transmittance at 1790 nm (marked with red circles) tends to a maximum value for ϑ=0° (ϑ=180°) and a minimum value for ϑ=90° (ϑ=270°). The polarization-dependent transmittance with a two-lobed shape indicates the linear dichroism effect [60] and provides the ability to enact the double FRs-based plasmonic switching in Figure 3a. With respect to the in-plane mirror symmetries but with rotational asymmetries (that is, the center offset distance is 15 nm and α=2β=40°), the transmittance of all four wavelengths exhibits periodic dependence on the polarization angles with a two-lobed shape and a period of 180°, triggering two pairs of polarization-resolved dual-wavelength plasmonic switching that is shown in Figure 6a. In Figure 7c, considering the effect of the mirror and rotational asymmetries (namely set the center offset distance as 15 nm and the asymmetric angle β=40°), the transmittance of all four wavelengths show distinct polarization effects that unfold a two-lobed shape and a periodicity of 180°, as evident by the two-fold symmetry. Therein, the transmittance of 1083 nm (marked with red circles) is maximized when the incident light is longitudinally polarized (90° and 270°), whereas the transmittance at 1010 nm (marked with black squares) supports two maxima at 100° and 280°, thereby resulting in the presence of high ON/OFF ratios being 20.93 dB and 17.32 dB, respectively. Also, the transmittance peaks of 1201 nm and 1836 nm (marked with green up-triangle and blue down-triangle) display two maxima at 120° (300°) and 10° (190°), respectively. These behaviors imply the strong polarization effects with in-plane anisotropic properties and offer a great flexibility to unveil the polarization-resolved optical properties and device applications.

## 7. Conclusions

In summary, we have theoretically and numerically studied the near-infrared multiple FRs for the realization of multi-wavelength passive plasmonic switching in a novel hybrid metasurface, which consists of periodic Ag film arrays with DSRA and CRA unit cells. It is shown that the double FRs-based transmittance, which emanates from the interaction among the double bonding mode, double anti-bonding mode, and degenerate resonance mode, can be exploited for dual-wavelength plasmonic switching with the ON/OFF ratios being 28.81 dB and 20.76 dB, respectively. Following that, by varying the width of DSRA ($W_{1}$) and CRA ($W_{2}$) unit cells, the customized dual-wavelength plasmonic switching can be realized with high ON/OFF ratios, strong modulation depths, and the resonance center wavelengths being redshift or blueshift dependent. By breaking the in-plane mirror symmetries of the DSRA unit cells, the multiple FRs-based triple-wavelength plasmonic switching can be fulfilled with high ON/OFF ratios and strong modulation depths. Concerning the impact of breaking C2 in-plane rotational symmetries, the multi-wavelength plasmonic switching can be implemented with one of the double dual-wavelength plasmonic switches containing quasi-BICs-embedded modes, as well as tunable and broadband bandwidth (∼205 nm). Meanwhile, these behaviors in turn sustain that the degenerate resonance mode is plasmonic BIC mode. For the polar plots of the transmittance of the related switching points, the polarization-dependent transmittance with a two-lobed shape and a period of 180° uncovers the linear dichroism effects with in-plane anisotropic properties and enacts the multiple FRs-based multi-wavelength plasmonic switching. Finally, our results offer a new perspective toward realizing multi-wavelength selective passive plasmonic switching that is useful for optical communication, information processing, and sensing systems.

## Data Availability

The data presented in this study are available on request from the first or corresponding author.
